# Decreasing Seven-Day Revisits After Pediatric Tonsillectomy: A Multidisciplinary and Comprehensive Unit-Based Safety Program (CUSP)-Driven Quality Improvement Initiative

**DOI:** 10.7759/cureus.100068

**Published:** 2025-12-25

**Authors:** Chris Deskins, Helene Dabbous, Samantha Smith, Paul Bryan Hankey, Olivia Rickenbach, William S Jones, Kayla Kessler, Jessica Lawrence, Candy A Mahle, Pavithra Ellison

**Affiliations:** 1 Anesthesiology, West Virginia University, Morgantown, USA; 2 Otolaryngology, West Virginia University, Morgantown, USA; 3 Quality, WVU Medicine, Morgantown, USA; 4 Perioperative Services, WVU Medicine, Morgantown, USA

**Keywords:** comprehensive unit-based safety program, pain optimization, perioperative dehydration prevention, post-discharge revisit, tonsillectomy

## Abstract

Introduction: Pediatric tonsillectomy is among the most common ambulatory procedures in children. An important aspect of serving this patient population is appropriate post-operative care and follow-up. In early 2025, our institution identified a seven-day revisit rate in post-tonsillectomy patients exceeding those of similar institutions nationally. We identified possible areas of improvement, including improved hydration, post-operative pain management, and clear instructions for post-operative care.

Objective: Our goal was to reduce the seven-day revisit rate ≥1% by December 31, 2025, for patients ages 0-21, through the implementation of a perioperative dehydration-prevention and pain-optimization bundle. This intervention included an intraoperative medication protocol, standardized discharge materials, and communication optimization within a Comprehensive Unit-based Safety Program (CUSP) framework.

Methods: This study was conducted at WVU Medicine Golisano Children's Hospital in Morgantown, West Virginia. A multidisciplinary team, including anesthesiology, otolaryngology, nursing, and quality-trained staff, applied three sequential Plan-Do-Study-Act (PDSA) cycles under CUSP governance. Our intraoperative pathways involved administration of a crystalloid bolus ≥30 ml/kg (max 1.5 L), as well as acetaminophen (15 mg/kg, max 1000 mg) and dexamethasone (0.5 mg/kg, max 20 mg) dosing. Goals for our Post-Anesthesia Care Unit (PACU) encompassed scheduled ibuprofen dosing 10 mg/kg (max 400 mg), standardized discharge packets containing a 48-hour hydration checklist and dye-free medication calendar, as well as planned check-ins and updates with family by the surgical team. We performed monthly reviews of revisit rates along with process and balancing measures.

Results: Early into implementation, our interventions identified pain and dehydration as predominant factors for revisits. As the study progressed, we observed that our adopted interventions led to standardized intraoperative fluids and analgesia, improved discharge clarity, and aligned communication workflows with caregiver expectations.

Conclusions: A pragmatic perioperative-to-home bundle focused on hydration, multimodal analgesia, and practical discharge instructions showed early feasibility for reducing short-term revisits (3.75-2.16%). Longitudinal monitoring will evaluate outcome durability and assess the sustainability of improvements.

## Introduction

Tonsillectomy, with or without adenoidectomy, remains a cornerstone of pediatric otolaryngologic care. However, this surgery carries substantial post-discharge morbidity, particularly from pain, dehydration, and postoperative nausea and vomiting (PONV) [1,2,3]. These complications, when poorly controlled, have been associated with higher revisit and readmission rates [2,3,4]. Improper post-operative care due to unclear and incomplete discharge instructions has also been shown to be a contributing factor to higher revisit rates [2,4]. National readmission data indicate 30-day emergency department revisit rates of approximately 10%, with readmissions near 6% [5]. These events contribute to unwanted caregiver anxiety, patient distress, and increased healthcare utilization. 

At our institution, an internal audit of data from January through December 2024 demonstrated a revisit rate of 3.75%. This rate exceeds Vizient's national top-quartile benchmark rate of 2.17%. Vizient Inc. (vizientinc.com) is a member-owned healthcare performance improvement company in the United States. Most hospitals and academic medical centers use Vizient for benchmarking, quality improvement, supply chain optimization, and operational analytics. A qualitative review of reports and caregiver feedback from Press Ganey (pressganey.com) institutional data highlighted variable intraoperative hydration administration, inconsistent analgesia practices (both administration and dosing of opioids and ibuprofen), and the perception of rushed discharge with unclear follow-up instructions. In response, a multidisciplinary CUSP team developed an evidence-based perioperative dehydration and pain prevention bundle [6]. 

## Materials and methods

Design and setting  

Our quality initiative project was a prospective, multidisciplinary quality improvement project at a single tertiary children’s hospital (WVU Medicine Golisano Children's Hospital in Morgantown, West Virginia) using sequential PDSA cycles under a CUSP governance model. The team included anesthesiology, otolaryngology, preoperative, and PACU nursing, along with the Center for Quality Outcomes. The target for achieving a 1% reduction in the revisit rate was set for December 2025.

We requested a determination letter from our institutional review board (Office of Research and Subject Protection) to conduct this quality improvement project and received approval to conduct the project. 

All ambulatory tonsillectomy patients ages 0-21 (with or without adenoidectomy) were included. Indications for surgery encompassed recurrent tonsillitis, sleep-disordered breathing (SDB), and obstructive sleep apnea (OSA). Exclusions included planned admissions, pre-specified complex airway cases such as Pierre Robin Sequence, and patients with congenital heart disease. PDSA cycles were implemented, and data from all included patients were collected retrospectively after each PDSA cycle.

The project launched on March 1, 2025. Retrospective data were extracted from Vizient Inc. to establish our baseline seven-day revisit rate for January through December 2024. Monthly revisit data were obtained from Vizient for March through October 2025. Of note, Vizient uses its proprietary algorithm to provide us with benchmarking data for comparison for our cohort of academic medical centers and has a two-month lag period.

Procedure description

Tonsillectomy with electrocautery under general anesthesia is the technique used in our institution by all surgeons involved in this initiative. Electrocautery provides simultaneous tissue division and hemostasis, which reduces intraoperative blood loss by sealing small vessels encountered during the dissection. Patients are intubated and positioned supine, and the oropharynx is exposed using a self-retaining mouth retractor. After adequate visualization is established, the surgeon gently grasps the superior pole of the tonsil and uses electrocautery to incise the mucosa and enter the plane between the tonsillar capsule and the underlying pharyngeal musculature. Dissection is carried out along this avascular plane from the superior pole to the inferior pole, which allows the tonsil to be removed in a controlled fashion.

Once the tonsil is removed, the tonsillar fossa is inspected thoroughly, and hemostasis is achieved using monopolar or bipolar cautery as needed. The same technique is used to remove the contralateral tonsil. At the conclusion of the procedure, the oropharynx is irrigated, and a final assessment is performed. If there is no evidence of active bleeding, the retractor is removed, and the patient is awakened from anesthesia.

Outcome and process measures  

The primary outcome measured was seven-day revisit rates. This was defined as any unplanned emergency department visits, as well as any urgent-care, observation, or inpatient encounters within seven calendar days of discharge related to dehydration, pain, bleeding, nausea/vomiting, fever, or failure to follow discharge instructions. 

​​Secondary outcomes observed included process measures (as a percentage for both intraoperative and postoperative care) and balancing measures. Intraoperative measures included patients receiving ≥30 ml/kg intraoperative crystalloid as well as patients receiving acetaminophen and dexamethasone. Postoperative measures evaluated patients with scheduled PACU ibuprofen, caregiver packet completions, and documented Physician PACU check-ins.​ Balancing measures included PACU length-of-stay, unplanned overnight admission, PONV rescue rate, and staff satisfaction [6].  

PDSA cycles and interventions  

Our first PDSA cycle (Intraoperative Standardization) ran from March to May of 2025 and focused on an intraoperative standardization bundle. This included ≥30 mL/kg crystalloid (max 1.5 L), acetaminophen 15 mg/kg (max 1000 mg), and dexamethasone 0.5 mg/kg (max 20 mg). Options for intraoperative morphine for pain control and dexmedetomidine for smoother emergence were available, with balancing measures monitoring sedation and PONV [3,7,8]. 

Our second PDSA cycle (PACU Practices and Early Analgesia), which is focused on recovery in the PACU, occurred from May 2025 to September 2025. This portion focused on continued IV hydration to achieve 30 ml/kg or greater, as well as a single 10 mg/kg dose (max 400 mg) of ibuprofen. For fluid intake beyond the targeted IV dose of 30 ml/kg, an adoption of tolerance-based hydration readiness criteria rather than fixed-volume requirements was implemented. We also instituted a caregiver survey to assess comfort with their PACU stay and messaging of care [9].

Our third PDSA cycle (Communication and Discharge Materials) began in September 2025 and is ongoing. It involves standardized communication workflows for caregiver updates and PACU check-ins by members of the surgical team, as well as preoperative scripting regarding nasal trumpet expectations while recovering in PACU. Parents will also be provided a redesigned discharge packet that contains procedure-specific instructions as well as a 48-hour hydration checklist. It also contains a time-based medication calendar that alternates acetaminophen 15 mg/kg every six hours, as well as ibuprofen 10mg/kg every six hours. Timing of these medications is coordinated with the most recent intraoperative and postoperative dosing in such a way that the patient is scheduled to receive pain medication every three hours to minimize episodes of uncontrolled pain. Parents are also provided a prescription for dye-free flavored versions of the medications. Of note, our institution does not routinely prescribe opioids at discharge for this patient population [4].

For a concise summary of our interventions and further details regarding discharge optimization, please refer to the appendix.

Analysis  

We performed monthly reviews for process and outcome measures as well as monthly chart audits for current revisit rates. Primary and secondary outcome measures were tracked and discussed at our CUSP monthly meetings. Any issues regarding implementation or roadblocks, as well as feedback from all stakeholders, were discussed and addressed. 

## Results

Pre-intervention

Our institutional review, evaluating a total of 1,041 patients between January 2024 and December 2025, noted a seven-day revisit rate of 3.75%. Pain and dehydration were found to be predominant revisit drivers for these encounters. Upon chart audit, notable highlights included variability in fluid administration (as low as 10 ml/kg) and inconsistency with ibuprofen dosing in PACU. A salient caregiver complaint cited perceived rushed care, inconsistent hydration targets, absence of a postoperative physician check-in, and a red-dye contradiction in take-home medications. Further discussion with PACU nursing staff corroborated inconsistent discharge messaging for post-tonsillectomy patients.

Post-intervention

**Figure 1:**
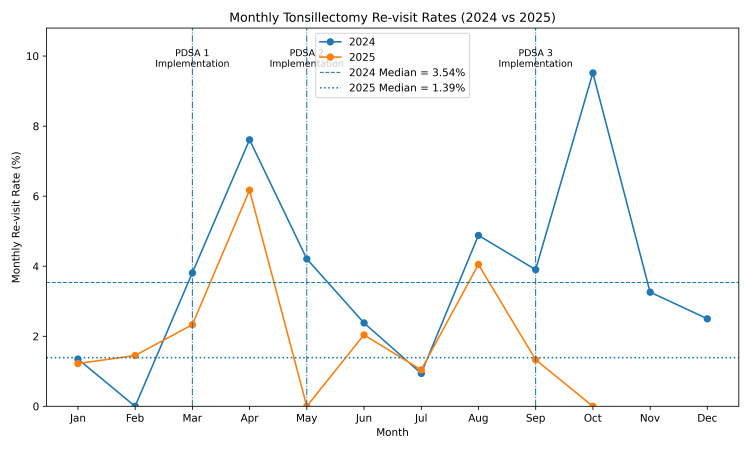
Seven-day re-visit rates in 2024 and 2025. Pre-intervention January 2024-February 2025. PDSA Cycle 1 initiated in March 2025.  PDSA Cycle 2 initiated in May 2025.  PDSA Cycle 3 initiated in September 2025. Data adapted from the Vizient database (vizientinc.com)

Following our interventions, a review of the data from March 2025 to October 2025 demonstrated an overall seven-day revisit rate of 2.16% (n = 15). This is a decreased revisit rate of 1.59% from our recorded baseline of 3.75%. Upon chart audit, pain was found to be the predominant revisit driver post-intervention. Figure 1 illustrates our monthly revisit rates before and after each stage of intervention. Table 1 lists associated ICD-10 codes for each revisit and highlights pain as the predominant factor necessitating evaluation. Table 2 depicts the improvement in revisit rates observed with each PDSA cycle. 

**Table 1:** Baseline patient counts and seven-day revisit rates by PDSA cycle PDSA: Plan-Do-Study-Act

PDSA cycle	Date range	Revisit (n)	7-day revisit rate (%)	Primary reasons
Pre-Intervention (n = 1041)	January–December 2024	39	3.75	Pain, dehydration
PDSA 1 (n = 247)	March–May 2025	7	2.83	Pain, dehydration
PDSA 2 (n = 423)	May–September 2025	7	1.65	Pain, dehydration
PDSA 3 (n = 133)	September–Current	1	0.75	Pain, dehydration

**Table 2:** ICD 10 codes for re-visit/re-admit for 2024 and 2025

2024	
ICD 10 Revisit Reason Codes	Patient Count
e860 - dehydration	5
g8918 - other acute postprocedural pain	16
h6691 - otitis media, unspecified, right ear	1
j029 - acute pharyngitis, unspecified	1
j189 - pneumonia, unspecified organism	1
j95830 - postproc hemor of a resp sys org fol a resp sys procedure	1
m436 - torticollis	1
n898 - other specified noninflammatory disorders of vagina	1
r0689 - other abnormalities of breathing	1
r109 - unspecified abdominal pain	1
r1110 - vomiting, unspecified	2
r112 - nausea with vomiting, unspecified	1
r509 - fever, unspecified	1
r638 - other symptoms and signs concerning food and fluid intake	2
t819xxa - unspecified complication of procedure, initial encounter	1
z9089 - acquired absence of other organs	1
z98890 - other specified postprocedural states	2
Grand Total	39
2025	
ICD 10 Revisit Reason Codes	Patient Count
e860 - dehydration	1
g8918 - other acute postprocedural pain	7
h9201 - otalgia, right ear	1
r040 - epistaxis	1
r059 - cough, unspecified	1
r1110 - vomiting, unspecified	1
r112 - nausea with vomiting, unspecified	2
r509 - fever, unspecified	1
s0990xa - unspecified injury of head, initial encounter	1
Grand Total	16

## Discussion

Results from this initiative are consistent with previously published work identifying pain and dehydration as the leading drivers of post-tonsillectomy revisits [10]. Large cohort studies have reported revisit or readmission rates between 0% and 15% within seven days, with inadequate oral intake and poorly controlled pain accounting for a substantial portion of early encounters [11]. Compared with these values, our preliminary post-intervention rate of 2.16% suggests a directionally favorable trend, although longer-term data and additional PDSA cycles are needed. 

The bundle elements used in this project (multimodal, opioid-sparing analgesia; structured PACU hydration readiness criteria; and caregiver updates with anticipatory guidance and preparations for procedure-specific discharge packets) mirror components shown in prior studies to improve postoperative comfort, reduce nausea/vomiting, and support early intake [12]. By integrating these evidence-supported practices within a CUSP framework, this initiative complements existing QI literature and provides a pragmatic, replicable approach for pediatric surgical services aiming to reduce preventable revisits. 

This initiative targeted multiple failure points along the perioperative continuum, contributing to dehydration and pain-related revisits as well as poor adherence to post-operative instructions. The intraoperative bundle provides highly reliable physiological support (adequate crystalloid loading and anti-inflammatory/analgesic coverage) while consolidating optional elements through criteria, consistent with prior findings [1,3,9]. In PACU, shifting from a fixed-volume rule to functional hydration readiness addresses early gaps in comfort and intake, reflecting evidence that tolerance-based approaches better predict safe discharge [13].

Regarding post-PACU care and follow-up, discharge redesign resolves messaging contradictions and equips families with a simple, time-specific plan and dye-free medications, aligning with evidence linking clarity to improved outcomes [4,14]. Communication standards directly respond to caregiver concerns, bolstering trust, readiness, and adherence to instructions [15]. We suspect much of the improvement shown in our follow-up interventions was due to improved dispensing instructions and adherence for alternating, scheduled doses of acetaminophen and ibuprofen. Studies have demonstrated not only that these medications can offer adequate pain control for most post-tonsillectomy patients, but also that they’re safe concerning post-operative bleeding and total daily dosing [16,17]. While the majority of studies evaluating alternating dosing regimens have focused on control of fever, some have suggested improved pain control at both three and four-hour dosing intervals [18,19,20].

Anticipated challenges include balancing opioid strategies with PONV and sedation risks, supporting nurse comfort with scheduled versus PRN orders, and preventing undue increases in PACU length of stay. Balancing measures and iterative PDSA cycles are designed to detect and mitigate these tradeoffs.  

Limitations identified include single-center design, early implementation (which may limit inference about effectiveness), and relevance to patients requiring planned admission (<3 years of age, severe OSA, coexisting disease, etc.). Further studies are warranted to look at longitudinal data and closely monitor strategies such as oxycodone use. The absence of robust statistical analysis of longitudinal data is also a limitation. Lastly, surgical technique: although electrocautery offers efficient dissection and reliable intraoperative hemostasis, the associated thermal injury to surrounding tissues may contribute to increased postoperative pain compared with cold dissection techniques. Nevertheless, electrocautery tonsillectomy remains the surgical technique utilized in this cohort of patients. 

## Conclusions

Unplanned revisits and readmissions for pediatric tonsillectomies are a stress to families and patients, as well as a burden to our healthcare system. A perioperative bundle emphasizing fluids, multimodal analgesia, hydration readiness, discharge clarity, and targeted communication, implemented via CUSP with PDSA cycles, is a practical pathway to reduce seven-day revisits after pediatric tonsillectomy. The next steps are continued implementation, sustained adherence, and outcome analysis. The focus of this article is to understand the value and potential efficiency of a multidisciplinary team working together to rapidly implement changes and improve measured outcomes. 

## Appendices

Bundle v1.0 (default unless contraindicated)  

OR: crystalloid 30 ml/kg; acetaminophen 15 mg/kg; dexamethasone 0.5 mg/kg (max 20 mg); optional dosing of morphine and dexmedetomidine with balancing measures.  

PACU: complete IVF; scheduled ibuprofen 10 mg/kg (max 400 mg); hydration readiness criteria with sip protocol and rescue pathway.  

Discharge: (Ongoing) three procedure-specific packets; 48-hour hydration checklist; dye-free, flavored medications; dosing calendar keyed to discharge time.  

Communication: (Ongoing) Surgeon PACU check-in; 20-minute status updates; pre-op nasal trumpet scripting. 

Discharge instructions improvements

Under Implementation

To improve caregiver comprehension, reduce return visits, and standardize postoperative care, we developed an enhanced set of discharge instructions. A single point of contact for questions or concerns was provided, along with signs and symptoms necessitating a phone call for further evaluation.  These symptoms included uncontrolled pain, poor oral intake, and  fevers ≥ 101.5°F (38.6°C)

We also added procedure-specific instructions (tonsillectomy alone vs tonsillectomy and adenoidectomy), guidance on assessing hydration status and when to call 911, and a built-in, weight-based medication timing table for alternating acetaminophen and ibuprofen every three hours (see Table 3).

**Table 3:** Time, medication, and dosage instructions

Time	Medication	Dose
6:00 AM	Acetaminophen	____ mg
9:00 AM	Ibuprofen	____ mg
12:00 PM	Acetaminophen	____ mg
3:00 PM	Ibuprofen	____ mg
6:00 PM	Acetaminophen	____ mg
9:00 PM	Ibuprofen	____ mg
12:00 AM	Acetaminophen (optional nighttime dose)	____ mg

Example Medication Schedule (Adjusted as Appropriate)

Key instructions included:

Do not exceed maximum daily doses:

Acetaminophen: 75 mg/kg/day (max 4,000 mg/day, single dose 15 mg/kg, max 1000 mg)

Ibuprofen: 30 mg/kg/day (max 2,400 mg/day, single dose 10 mg/kg, max 400 mg)

Avoid aspirin or products containing aspirin.

Parents are reminded to:

Use a medication log, included in the discharge paperwork.

Avoid duplicate dosing from over-the-counter cold/flu combinations.

## References

[1] Mitchell RB, Archer SM, Ishman SL (2019). Clinical practice guideline: tonsillectomy in children (update). Otolaryngol Head Neck Surg.

[2] Gan TJ, Belani KG, Bergese S (2020). Fourth consensus guidelines for the management of postoperative nausea and vomiting. Anesth Analg.

[3] Czarnetzki C, Elia N, Lysakowski C (2008). Dexamethasone and risk of nausea and vomiting and postoperative bleeding after tonsillectomy in children: a randomized trial. JAMA.

[4] LeClair AM, Sweeney M, Yoon GH, Leary JC, Weingart SN, Freund KM (2019). Patients' perspectives on reasons for unplanned readmissions. J Healthc Qual.

[5] Mahant S, Richardson T, Keren R, Srivastava R, Meier J (2020). Variation in tonsillectomy cost and revisit rates: analysis of administrative and billing data from US children's hospitals. BMJ Qual Saf.

[6] Pitts SI, Maruthur NM, Luu NP (2017). Implementing the Comprehensive Unit-Based Safety Program (CUSP) to improve patient safety in an academic primary care practice. Jt Comm J Qual Patient Saf.

[7] Hession-Laband E, Melvin P, Shermont H, Murphy JM, Bukoye B, Amin M (2018). Reducing readmissions post-tonsillectomy: a quality improvement study on intravenous hydration. J Healthc Qual.

[8] Miller AL, McCarty JC, Bergmark RW (2021). Association of perioperative ibuprofen exposure with post-tonsillectomy bleeding requiring operative management. Int J Pediatr Otorhinolaryngol.

[9] Zhu M, Wang H, Zhu A, Niu K, Wang G (2015). Meta-analysis of dexmedetomidine on emergence agitation and recovery profiles in children after sevoflurane anesthesia: different administration and different dosage. PLoS One.

[10] Johnson RF, Chang A, Mitchell RB (2018). Nationwide readmissions after tonsillectomy among pediatric patients - United States. Int J Pediatr Otorhinolaryngol.

[11] Alsuhebani M, Walia H, Miller R, Elmaraghy C, Tumin D, Tobias JD, Raman VT (2019). Overnight inpatient admission and revisit rates after pediatric adenotonsillectomy. Ther Clin Risk Manag.

[12] Kaan MN, Odabasi O, Gezer E, Daldal A (2006). The effect of preoperative dexamethasone on early oral intake, vomiting and pain after tonsillectomy. Int J Pediatr Otorhinolaryngol.

[13] (2021). Resources and tools to improve discharge and transitions of care and reduce readmissions. Resources.

[14] Navanandan N, Schmidt SK, Cabrera N, Topoz I, DiStefano MC, Mistry RD (2020). Seventy-two-hour return initiative: improving emergency department discharge to decrease returns. Pediatr Qual Saf.

[15] Xu V, Aaronson NL (2025). Reduced returns to ED with post-tonsillectomy and adenoidectomy chat program: outcomes and costs. Laryngoscope.

[16] Liu C, Ulualp SO (2015). Outcomes of an alternating ibuprofen and acetaminophen regimen for pain relief after tonsillectomy in children. Ann Otol Rhinol Laryngol.

[17] Bedwell JR, Pierce M, Levy M, Shah RK (2014). Ibuprofen with acetaminophen for postoperative pain control following tonsillectomy does not increase emergency department utilization. Otolaryngol Head Neck Surg.

[18] Mast G, Henderson K, Carr MM (2020). The effect of ibuprofen dosing interval on post-tonsillectomy outcomes in children: a quality improvement study. Ann Otol Rhinol Laryngol.

[19] Ong CK, Seymour RA, Lirk P, Merry AF (2010). Combining paracetamol (acetaminophen) with nonsteroidal antiinflammatory drugs: a qualitative systematic review of analgesic efficacy for acute postoperative pain. Anesth Analg.

[20] Shortridge L, Harris V (2007). Alternating acetaminophen and ibuprofen. Paediatr Child Health.

